# Identification of pesticides associated with an increased risk of Parkinson’s disease using a multi-screen approach

**DOI:** 10.1016/j.envint.2026.110087

**Published:** 2026-01-20

**Authors:** Marisol Arellano, Lisa M. Barnhill, Aaron M. Kim, Kazi Md Mahmudul Hasan, Sharon Li, Kimberly C. Paul, Chao Peng, Beate Ritz, Jeff M. Bronstein

**Affiliations:** a Molecular and Environmental Toxicology Interdepartmental Program, University of California Los Angeles, Los Angeles, CA, United States; b Department of Neurology, David Geffen School of Medicine, United States; c Department of Epidemiology, Fielding School of Public Health, University of California Los Angeles, Los Angeles, CA, United States

**Keywords:** Parkinson’s disease, Pesticides, Autophagy, α-Synuclein

## Abstract

Parkinson’s disease (PD) is a progressive neurodegenerative disease characterized by aggregation and transmission of alpha-synuclein (α-syn) protein and loss of dopaminergic neurons. The etiology of PD is multifactorial, involving both genetic and environmental factors. Pesticide exposure has been associated with PD, and with thousands of registered pesticides in the United States, it is still unclear which of these chemically and structurally diverse pesticides confer this association. The population-based case-control Parkinson’s, Environment, and Gene (PEG) study based in the California Central Valley, an agricultural hub servicing much of the nation, offers a promising opportunity to investigate this relationship and identify likely environmental risk factors contributing to PD risk.

In this study, 62 pesticides with reported agricultural use in the Central Valley were independently evaluated in 2 cell-based assays testing for pesticides that promote α-syn transmission and alter autophagy. To further stratify and prioritize pesticide candidates, pesticides that were positive in the 2 cell-based screens (double hits) were filtered through a newly described pesticide-wide association analysis to agnostically identify relevant real-world exposures. Using these selection criteria, 6 pesticides were identified as triple hits and were tested for dopaminergic neurotoxicity in an *in vivo* zebrafish (ZF) model. Of these 6 pesticides, 4 pesticides contributed to aminergic neuron loss in ZF larvae. The majority of the pesticides identified in our screens have not previously been implicated as risk factors for PD but should be considered in future studies.

## 1. Introduction

Parkinson’s disease (PD) is an increasingly prevalent neurodegenerative disorder globally, and the number of cases is expected to grow with the aging population (Collaborators GBDN, May 2019; Dorsey et al., 2018). The hallmarks of PD include pathological aggregation of alpha-synuclein (α-syn) protein in Lewy bodies, progressive loss of dopaminergic neurons in the substantia nigra, and neuroinflammation that lead to progressive motor and cognitive impairments (Spillantini et al., 1997; Jucker and Walker, 2013; Kannarkat et al., 2013; Lull and Block, Oct 2010). The etiology of PD is complex and involves multiple factors, including genes and the environment. The earliest pathology of aggregated α-syn is found in the gut and olfactory bulb and is believed to spread *trans*-synaptically throughout the central nervous system (Braak and Del Tredici, 2009; Peng et al., Apr 2020).

The α-syn protein plays an important role in the regulation of synaptic vesicle trafficking and fusion at the presynaptic terminal, but some aggregated forms of α-syn are toxic to neurons. Furthermore, insoluble α-syn has been shown to have seeding capability with monomeric α-syn, which can result in the spread of pathological α-syn species (Wu et al., 2019). Studies evaluating the transmissibility of pathogenic species of α-syn protein suggest that the intercellular spread of these pathological seeds precedes the templated amplification of misfolded α-syn in recipient cells to form misfolded protein inclusions that are resistant to enzymatic breakdown (Peng et al., Apr 2020; Peng et al., May 2018).

It is not well understood what initiates α-syn aggregation, but the identification of rare genetic forms of PD have provided important clues into its pathophysiology. Increased expression of α-syn arising from gene multiplication is sufficient to cause PD (Ibáñez et al., 2004; Miller et al., May 2004; Chartier-Harlin et al., 2004; Abeliovich et al., Jan 2000; Chen et al., 2013; Burré et al., 2010). Furthermore, polymorphisms in the α-syn gene (*SNCA*) are associated with increased risks of developing PD and faster symptom progression in those who develop PD (Chiba-Falek and Nussbaum, Dec 2001; Mata et al., Nov 2010; Ritz et al., 2012). Other gene mutations leading to PD have implicated disruption of proteostasis as an adverse outcome pathway in at least some familial cases (Chartier-Harlin et al., 2004; Lesage and Brice, 2009; Kalinderi et al., Nov 2016; Ngo et al., 2024). For example, mutations in *GBA*, *LRRK2*, *VPS35*, *RAB32*, and *ATP13A2* have been identified as genetic risk factors for familial PD, leading to dysfunctional autophagy and impairing a primary mechanism for clearing α-syn aggregates. Further support for altered autophagy as a pathogenic pathway to developing PD comes from environmental studies. Air pollution has recently been identified as a risk factor for PD and diesel exhaust extracts have been reported to produce PD pathology by disrupting autophagy (Jami et al., 2021; Barnhill et al., 2020; Ha et al., 2022). Thus, increased levels of α-syn either through increased expression or reduced degradation appear to contribute to the development of PD.

Since genetic factors alone account for a minority of PD cases, it is likely that environmental factors also contribute considerably to PD risk. As the environment is modifiable, it is imperative to identify specific environmental factors that alter the risk of disease and determine the mechanisms by which they act. Pesticide exposure is one of the most well-documented environmental risk factors associated with PD (Brown et al., Feb 2006; Wang et al., Jul 2011; Kamel et al., Feb 2007). Pesticides are chemicals used to control, repel, or kill pests, such as insects, rodents, fungi, and weeds. They are widely used in the United States, both commercially and privately, and are composed of hundreds of different chemicals (Kamel et al., Feb 2007; Ball et al., 2019; Goldman, 2014). Several studies have reported the association between pesticides and PD, but only a few specific chemicals have been identified that confer increased risk. In this study, we leverage a recently developed novel method to assess the relationship between nearly 300 individual pesticides and PD using an untargeted approach for pesticide exposures and population-based case-control study data (Paul et al., 2023).

**Fig. 1.**
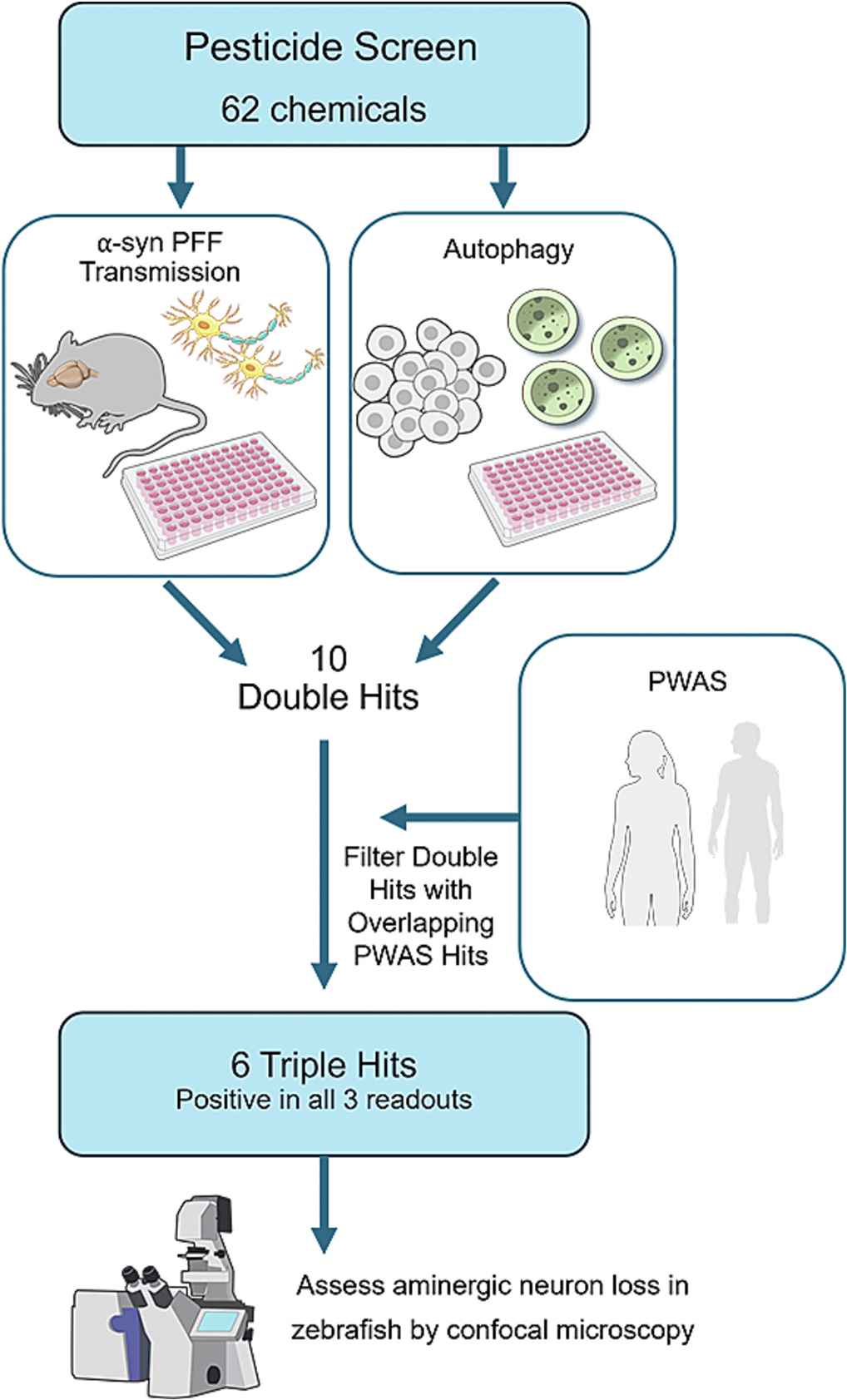
Pesticide Screen Schematic. An overview of the organization of the screen to evaluate candidate pesticides on key adverse outcome pathways involved in Parkinson’s disease (PD) and on individual chemical association with PD using a pesticide exposure-wide association study (PWAS). Pesticide candidates are funneled through each cell-based assay independently, and the pesticide candidates are filtered through the PWAS to identify hits that are positive in all 3 readouts. The hits are then evaluated for the effect on aminergic neuron loss in a ZF model of neurodegeneration. Abbreviations: ɑ-syn, alpha-synuclein; PFF, preformed fibril; PWAS, pesticide exposure-wide association study.

To now help determine whether pesticides identified epidemiologically are working through adverse outcome pathways (AOPs) associated with PD, we used 2 cell-based screens to identify pesticides that promote α-syn transmission and alter autophagy. Filtering the overlapping results of the cell-based screens through an epidemiologic pesticide exposure-wide association study (PWAS), we prioritized pesticides with mechanistic links to PD. The intent of this approach is to identify pesticides most likely to be altering PD risk with the understanding that it will not be all-inclusive as there are likely other pathological pathways involved in the pathogenesis of PD. Still, through this approach, we aimed to identify specific pesticides that promoted α-syn transmission, altered autophagy, and were associated with PD in the PWAS to assess the biological plausibility of these exposures *in vivo* (Fig. 1).

## 2. Methods

### 2.1. Chemicals and reagents

Chemicals were primarily purchased from ChemService (West Chester, PA, USA) and Sigma-Aldrich (St. Louis, MO, USA). A full list of the chemicals can be found in Supplement Table 1. Information for reagents purchased for experiments is listed below. Pesticide stock solutions were visually inspected for solubility. Final dimethyl sulfoxide (DMSO, vehicle) concentration was maintained at 0.1% across all assays.

### 2.2. Pesticide selection

Pesticide selections for the cell-based screens were informed by reported agricultural use in California’s Central Valley and exposure to the patients and controls of Parkinson’s disease within the Parkinson, Environment, and Gene (PEG) study cohort. California law mandates the recording of all commercial agricultural pesticide applications, allowing us to track the individual pesticide active ingredients that have been applied near the homes and workplaces of the PEG study participants since 1974 (Paul et al., 2023). Overall, 722 different chemicals were applied within a 500 m buffer of at least one PEG participant’s residence or workplace. We aimed to agnostically assess chemicals in relation to PD and therefore included as many pesticides as possible while balancing for the labor-intensive assays conducted in the study. The pesticide screen panel was limited to pesticides for which we found that greater than 10% of the PEG study participants had been exposed (n = 137 pesticides), then from these, we selected a subset that included at least 1 pesticide from each chemical class. In total, 62 pesticides met these criteria (Supplement Table 1) and were incorporated into the pesticide panel to be screened.

### 2.3. Animal husbandry and care

Embryos from gestating CD1 mice (Charles River Laboratories, Thousand Oaks, CA, USA) were used for primary neuron cultures. Dams were maintained on a standard 12-hour light/ 12-hour dark cycle with *ad libitum* access to food and water.

Zebrafish (ZF, *Danio rerio*) were raised at 28°C in recirculating water tanks on a 14-hour light / 10-hour dark cycle and fed twice daily with brine shrimp. Embryos and larvae were obtained from natural mating and staged according to days post fertilization (dpf). ET*VMAT2*:eGFP ZF, which expresses eGFP in aminergic neurons under the vesicular monoamine transporter 2 (*VMAT2)* promoter, were used for neuronal quantification (Lulla et al., Nov 2016). All breeding, housing, and experimental procedures adhered to the NIH Guide for the Care and Use of Experimental Animals and were approved by the UCLA Institutional Animal Care and Use Committee (IACUC).

### 2.4. Fibril preparation

Purification of recombinant human α-syn and generation of α-syn preformed fibrils (PFFs) were performed as previously described (Volpicelli-Daley et al., Sep 2014). Briefly, the pRK172 plasmid containing the mouse *SNCA* gene was transformed into BL21 (DE3) competent E. *coli* (C2527H, New England). A single colony was expanded in Terrific Broth (12 g/L Bacto-tryptone, 24 g/L yeast extract, 4 mL/L glycerol, 9.4 g/L KH_2_PO_4_, and 2.2 g/L K_2_HPO_4_) supplemented with ampicillin. Bacterial pellets were collected, sonicated, and boiled to precipitate unwanted proteins. The supernatant was dialyzed overnight in 10 mM Tris (pH 7.6), 50 mM NaCl, and 1 mM EDTA. The protein solution was then filtered through a 0.22 μm filter and concentrated using Pierce Protein Concentrators (PI88517, Thermo Scientific). The concentrated protein was loaded onto a Superdex 200 column (28990944, Cytiva), and 1 mL fractions were collected. These fractions were analyzed by SDS-PAGE and Coomassie blue staining, and those highly enriched in α-syn were pooled and dialyzed overnight in 10 mM Tris (pH 7.6), 25 mM NaCl, and 1 mM EDTA.

The dialyzed protein was subjected to anion exchange chromatography using a HiTrap Q HP column (17115401, Cytiva) with a linear gradient from 25 mM to 1 M NaCl. Collected fractions were analyzed by SDS-PAGE and Coomassie blue staining, and those enriched in α-syn were pooled, dialyzed into DPBS, filtered through a 0.22 μm filter, and concentrated to > 5 mg/mL using Pierce Protein Concentrators. The purified monomer was aliquoted and stored at −80°C.

To generate α-syn PFFs, 5 mg/mL α-syn monomer was incubated at 37°C with shaking at 1,000 rpm for seven days.

### 2.5. Primary neuron isolation

Mouse hippocampal neurons were prepared in-house from E16.5 embryos of CD1 mice (Charles River) as previously described (Peng et al., May 2018; Volpicelli-Daley et al., Sep 2014; Zhang et al., Feb 2023). Prior to cell plating, 384-well plates (781091, Greiner Bio-One) were coated with 0.1 mg/mL Poly-D-lysine (P0899, Sigma-Aldrich) in 50 mM borate buffer (pH 8.5) and incubated overnight at room temperature. The following day, plates were washed five times with ddH_2_O before use. On the day of dissection, E16.5 embryos were extracted from the uterus and decapitated. The heads were rinsed four times in ice-cold 3 + HBSS (500 mL HBSS (21-021-CM, Gibco) supplemented with 1% 1 M HEPES (15630080, Gibco), 1% 100 mM sodium pyruvate (25000CI, Corning), 0.5% penicillin–streptomycin, and 100 mL ddH_2_O) and stored in the same ice-cold solution. Hippocampus tissues were dissected following euthanasia as previously described (Fath et al., 2009), and stored in ice-cold Hibernate E solution (NC0285514, Fisher Scientific) supplemented with 1% B-27 Plus (A3582801, Gibco) and 1% GlutaMAX. The hippocampus tissues were transferred to a tissue culture hood, washed repeatedly with sterile 3 + HBSS in a 15 mL conical tube, and digested with papain-containing HBSS solution (20 U/mL papain (LS003126, Worthington), 5 mM L-cysteine, 1.1 mM EDTA (pH 8.5)) at 37°C for 7–10 min. DNase I (LS006355, Worthington) was added halfway through digestion. The reaction was quenched with fetal bovine serum (FBS), followed by sequential washes with 3 + HBSS, Neuron Basal Medium (10888022, Gibco) and plating medium to remove residual enzymes.

Tissues were gently dissociated into a single-cell suspension by pipetting in 1 mL plating medium (complete neuronal medium (Neuron Basal Medium supplemented with 2% B-27 Plus, 1% GlutaMAX, and 1% penicillin–streptomycin) with an additional 5% FBS). The suspension was passed through a cell strainer to remove undissociated cells. Cells were counted, diluted in plating medium, and seeded at 7–9 × 10 (Spillantini et al., 1997) cells/well. After approximately 3 h, once cells had adhered to the well bottom, the plating medium was replaced with complete neuronal medium (without FBS) to prevent the overgrowth of non-neuronal cell types. Neurons were maintained in a humidified incubator at 37°C with 5% CO_2_.

### 2.6. α-syn PFF transmission assay

Cultured mouse primary hippocampus neurons were co-incubated with 10 μM pesticide and α-syn PFFs at 7 days *in vitro* (DIV). The α-syn PFF transmission assay was conducted as previously described (Volpicelli-Daley et al., Sep 2014). Prior to neuronal transduction, α-syn PFFs were diluted to the desired concentration in complete neuronal culture medium. The water bath sonicator (Diagenode) was pre-chilled to below 8°C and operated in high power mode. Diluted PFFs were sonicated for 20 cycles of 30 s on / 30 s off, maintaining the bath temperature at approximately 8°C throughout the procedure. Immediately after sonication, the PFF-containing medium containing 10 μM pesticide with a final DMSO concentration of 0.1% was applied to neuronal cultures for transduction. At seven days post treatment, half of the neuronal medium was replaced with fresh media. Primary neurons were treated for 14 days, fixed with 4% paraformaldehyde (PFA) and evaluated for both toxicity and the development of α-syn pathology as previously reported (Luna et al., Jun 2018). Briefly, neurons were fixed with 4% PFA/4% sucrose in PBS followed by permeabilization with 1% Triton X-100. To stain insoluble pathological synuclein, cells were incubated overnight at 4°C with phosphorylated α-syn antibody 81A (81A) (1:4000) in blocking buffer. Neuronal viability was assessed simultaneously by staining with neurofilament light (NFL) antibody (1:4000) and mature neuronal marker NeuN (1:2000) in blocking buffer. We determined that NFL provided a more sensitive and direct measure of neurotoxicity, while NeuN labeling demonstrated the presence of mature neurons in our primary cultures. Following primary antibody incubation, cells were washed with PBS and incubated for 1 h at room temperature with secondary antibodies: goat anti-mouse IgG2a 568 and goat anti-rabbit 488, both diluted 1:2000 in blocking buffer, along with 1 μg/mL DAPI for nuclear staining. Cells were washed again with PBS to remove unbound secondary antibodies and DAPI before imaging. Finally, 81A/NFL levels for each condition were normalized to the PFF-only condition. To minimize Type II errors (false negatives) due to biological variance in the α-syn transmission assay, we employed a Boolean consensus rule to identify hits. A pesticide was considered a “hit” if it exceeded the PFF reference baseline (>1) in at least 2 of 3 independent trials. Assay performance was monitored on a per-plate basis by evaluating the signal-to-noise ratio between the PBS (negative) and PFF-only (reference baseline) conditions. Only plates exhibiting established baseline pathology in the PFF-only reference wells were included in the analysis, ensuring functional consistency across replicates in the assay.

### 2.7. LIVE/DEAD assay in SK-N-MC cells

Human neuroblastoma SK-N-MC cells were grown and maintained at 37°C and 5% CO_2_ in DMEM with 10% FBS, 5% penicillin/streptomycin. To determine the highest nonlethal concentration (defined as inducing < 25% increased cell death relative to vehicle), SK-N-MC human neuroblastoma cells were treated with vehicle or 0.1 μM −30 μM concentrations of each pesticide for 24 h. Cell viability was determined as a ratio of ethidium homodimer-1 to calcein-AM fluorescent signal as read by microplate reader and normalized to vehicle-treated cells per manufacturer’s instructions for the Live and Dead Cell Assay (ab115347, Abcam, Waltham, MA, USA). The highest nonlethal concentration for most pesticides in the screen was 10 μM – 30 μM, with few exceptions. To standardize the screen, 10 μM was the concentration used unless otherwise noted.

### 2.8. Autophagy assay in live SK-N-MC cells

Human neuroblastoma SK-N-MC cells were grown at 37°C and 5% CO_2_ in complete DMEM (10% FBS, 5% penicillin/streptomycin). SK-N-MC cells were exposed to either vehicle or the highest nonlethal concentration of pesticide for 24 h at 37°C. Labelling of autophagosomes by Autophagy Detection Kit green detection reagent (ADK; ab129484, Abcam, Cambridge, UK) and lysosomes by Lysotracker Red DND-99 (LTR; L7528, Invitrogen). Chloroquine (autophagy inhibitor) served as positive control, and paired vehicle controls were included for each experimental well to account for changes in autophagy over the acquisition period. Briefly, after cells were exposed to vehicle or pesticide in complete DMEM for 24 h, cells were washed with 1X Assay buffer supplemented with 5% FBS. Cells were then incubated with 100 μL of Microscopy Detection Reagent (2 μL Green Detection Reagent/ 1 mL 1X Assay buffer, 5% FBS) with 1 μL Lysotracker for 30 min at 37°C protected from light. Cells were washed twice with 200 μL 1X Assay buffer, 5% FBS before 100 μL of 1X Assay buffer, 5% FBS was added to each well. SK-N-MC cells were immediately live-imaged using laser scanning confocal microscopy (LSCM, Leica Microsystems Inc, Buffalo Grove, IL, USA); 63x oil-immersion objective with 2x zoom was used to capture images for analysis.

### 2.9. Autophagosome and lysosome foci image analysis

Dye-labelled autophagosome and lysosome foci and cell counts were measured using ImageJ (National Institutes of Health, Bethesda, MD, USA) image processing software with “FociPicker3D” package (Du et al., Dec 2011). Briefly, vehicle- and positive control-treated samples were used to manually count foci to establish the threshold to accurately identify foci with FociPicker3D. The threshold was set uniformly across all images. Foci counts were normalized to the number of cell outlines counted manually in collapsed z-stack images. Autophagosome and lysosome foci/cell counts were further normalized to the paired vehicle-treated condition and statistical analyses were conducted on the dataset. Pesticides were considered a hit for altered autophagy if the pesticide significantly increased or decreased foci/cell counts (P < 0.05) as determined by a two-tailed Student’s T-Test.

### 2.10. PWAS analysis

As previously described, a PWAS epidemiologic screen of 288 pesticides was conducted, linking 53 to PD at a false discovery rate (FDR) < 0.05 and 68 at FDR < 0.10 (Paul et al., 2023). Briefly, data from 1653 participants of the PEG study (n = 829 PD patients and n = 824 controls) were used to test each pesticide for association with PD in an untargeted, agnostic manner. PEG is a population-based case-control study set in three agricultural counties in Central California (Kern, Fresno, and Tulare) (Ritz et al., Mar 2016). For each study participant, lifetime geocoded residential and workplace address histories were linked to the California’s pesticide use report (PUR) database, which documents 50 years of agricultural application of hundreds of pesticides. Ambient exposure to each pesticide was estimated based on residential and workplace proximity to commercial pesticide applications, for example due to living near farms applying pesticides. A geospatial algorithm was used which combines the PUR database with maps of land-use and crop cover to determine for each individual pesticide active ingredient in the PUR, the reported pounds of pesticide applied per acre within a 500 m buffer around specific locations, such as addresses, yearly since 1974. For each pesticide, in the PUR and each PEG participant, the average pounds of pesticide applied per acre per year within a 500 m buffer of each residential and workplace address over the study window (1974 to 10 years prior to index date, which was PD diagnosis for patients or interview date for controls) was determined. Each pesticide was assessed individually for PD risk in a PWAS analysis. More detail has been published (Paul et al., 2023). Here, we linked the results of this PWAS for each of the 62 pesticides included in the experimental screens to integrate output from the two cell-based screens, highlighting pesticides linked to PD through both AOPs, and prioritize pesticides for the *in vivo* analysis.

### 2.11. Pesticide treatment in ZF

Manually dechorionated 1 dpf *VMAT2:*eGFP ZF embryos were incubated with vehicle or pesticide (0.1 μM −10 μM) in E3 buffer [5 mM NaCl, 0.17 mM KCl, 0.33 mM MgSO_4_, 0.33 mM CaCl_2_,]) with a final concentration of 0.1% DMSO], at 28.5°C for 6 days. ZF were treated at a density of 2 larvae/mL, and typically no more than 15 larvae were treated per well. ZF were regularly monitored during the treatment period for significant morphological abnormalities and stress, and attrition due to treatment.

### 2.12. ZF brain image acquisition and neuron counts

ZF larvae were anesthetized with < 0.01% Syncaine (MS-222) (Syndel, Ferndale, WA, USA) and fixed in 4% PFA. Larvae were washed in PBS, antibody labeled, then cleared in 100% glycerol before ZF whole brain tissues were dissected and mounted onto coverslips for confocal imaging at 40x magnification on a Leica SPE. Briefly, larvae were incubated with peroxide (3% H2O2, 0.8% KOH in PBS) for 8 min to clear pigment, permeabilized with Proteinase K at 10 μg/mL for 12 min and blocked in 5% lamb serum/5% donkey serum in 0.1% Triton X-100 in PBS. Neurons were immunolabeled with primary monoclonal chicken anti-GFP antibodies (1:1000, A10262, Thermo Fisher Scientific), detected with goat anti-chicken Alexa Fluor 488 (1:1000, A11039, Thermo Fisher Scientific). Larvae were then washed and cleared in 100% glycerol. LSCM was used to image ZF larvae (7 dpf); 40x-oil immersion objective with 1x zoom was used to capture the telencephalic and diencephalic brain regions for analysis. Alexa Fluor 488 + neurons in *VMAT2*:eGFP ZF were counted using ImageJ (NIH). Images were randomized and Alexa Fluor 488 + positive neurons were counted in the telencephalic and diencephalic regions. Once the data set was counted, the images were unblinded and the data were collated and analyzed.

### 2.13. Statistical analysis

Primary neuron and ZF aminergic neuron data are represented as mean ± SEM. Foci counts are represented in a heatmap (log_2_ fold-change from vehicle-treated cells) and triple hit foci count tabular results are represented as mean ± SEM. Sample size (N) are provided per condition in each figure legend. Pair-wise comparisons for foci counts in SK-N-MCs were analyzed using Student’s two-tailed *t*-test. Dose response to pesticide in ZF was analyzed by one-way ANOVA with Dunnett’s post-hoc multiple comparisons test. Statistical significance was considered at P < 0.05; groups being compared are identified in the figure legend. Statistical analyses were conducted using GraphPad Prism v. 9.5.1 software (GraphPad, Boston, MA, USA).

## 3. Results

### 3.1. Identification of pesticides that promote α-syn PFF transmission

**Fig. 2.**
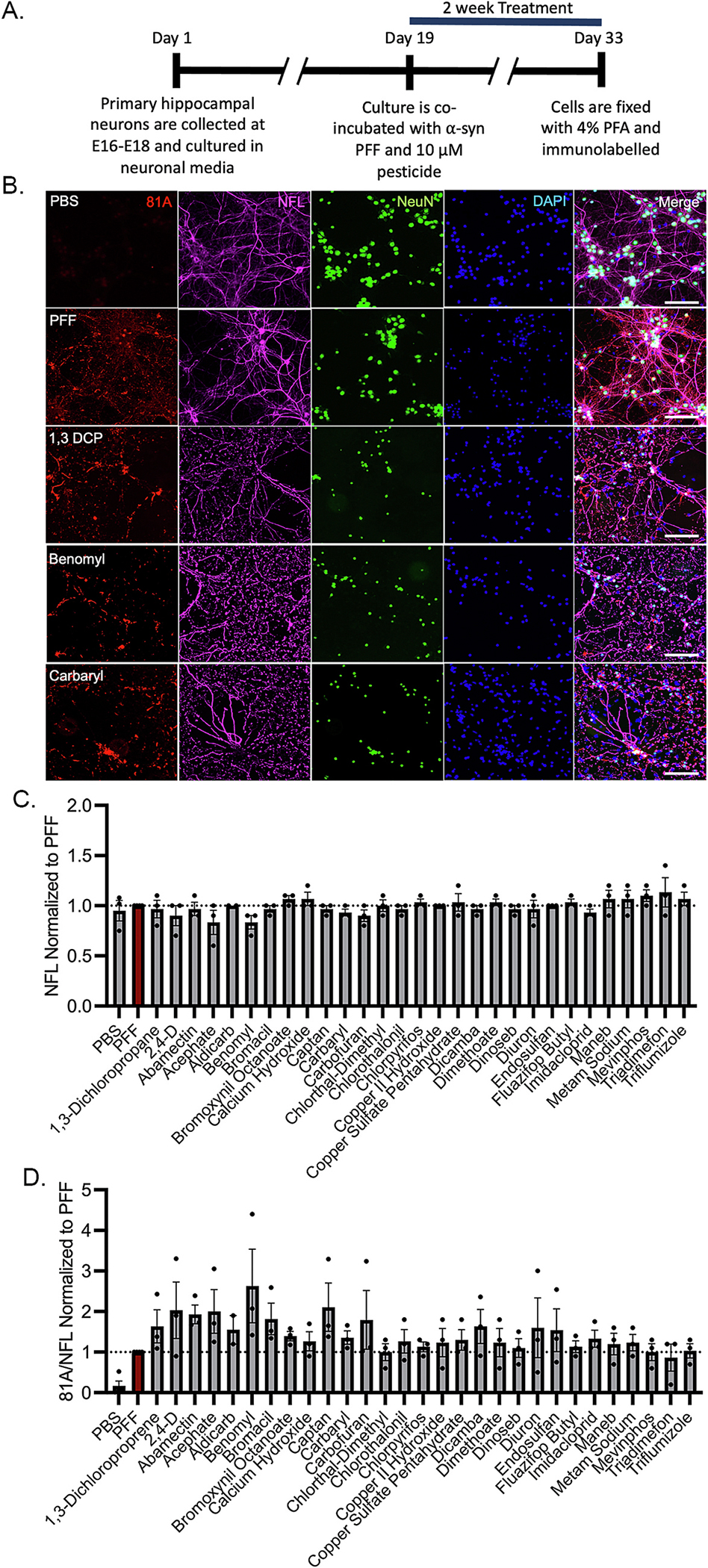
Pesticide-induced transmission of pathogenic alpha-synuclein pre-formed fibrils (ɑ-syn PFFs) in mouse primary hippocampal neurons. A) Treatment schema outlining the treatment timeline from collection, co-incubation, fixation, and immunolabelling of primary cultures. B) Primary cultures were treated for 14 days with PBS, PFFs, or PFFs and pesticide, fixed, and immunolabelled with phosphorylated ɑ-syn 81A (red, 81A), neurofilament light (magenta, NFL), NeuN (green, NeuN), and DAPI (blue). Scale bars: 50 μM. C) Pesticides were found to not have exhibit increased neuron loss relative to the PFF-only condition as shown by NFL levels. D) Twenty-eight pesticides show increased levels of 81A/NFL levels compared to PFF-only condition. Images shown are representative images for each condition. Data shown as mean ± SEM and is normalized to PFF-only condition; N = 3 experimental replicates. The pesticide was considered a hit if the 81A/NFL value was > 1 in 2 of 3 independent trials. Abbreviations: NFL, neurofilament light chain; ɑ-syn, alpha-synuclein; PFF, preformed fibrils; 1,3-DCP, 1,3-dichloroproprene. (For interpretation of the references to colour in this figure legend, the reader is referred to the web version of this article.)

The 62 pesticides were evaluated for their ability to promote pathogenic α-syn transmission in primary mouse neurons. Primary cultures were treated for 14 days with α-syn PFFs or α-syn PFFs and pesticide, fixed, and the amount of α-syn pathology was measured by indirect immunofluorescence with antibody against 81A, NFL, and NeuN (Fig. 2 A–B). Most of the pesticides did not exhibit increased toxicity at 10 μM compared to the PFF-only condition as measured by NFL labelling (Fig. 2C, Supplement Fig. 1A). Of the 62 pesticides screened, 29 pesticides showed an increased amount of 81A/NFL levels compared to the PFF-only condition (Fig. 2D, Supplement Fig. 1B), suggesting that these pesticides promote pathological α-syn transmission. The largest increases in pathological α-syn were found after exposure to benomyl, captan, acephate, abamectin, 2,4-D and bromacil in the presence of α-syn PFFs. Results for NFL and 81A/NFL levels for the full panel of pesticides in the screen are presented in Supplement Fig. 1.

### 3.2. Identification of pesticides that alter autophagy in SK-N-MC cells

**Fig. 3.**
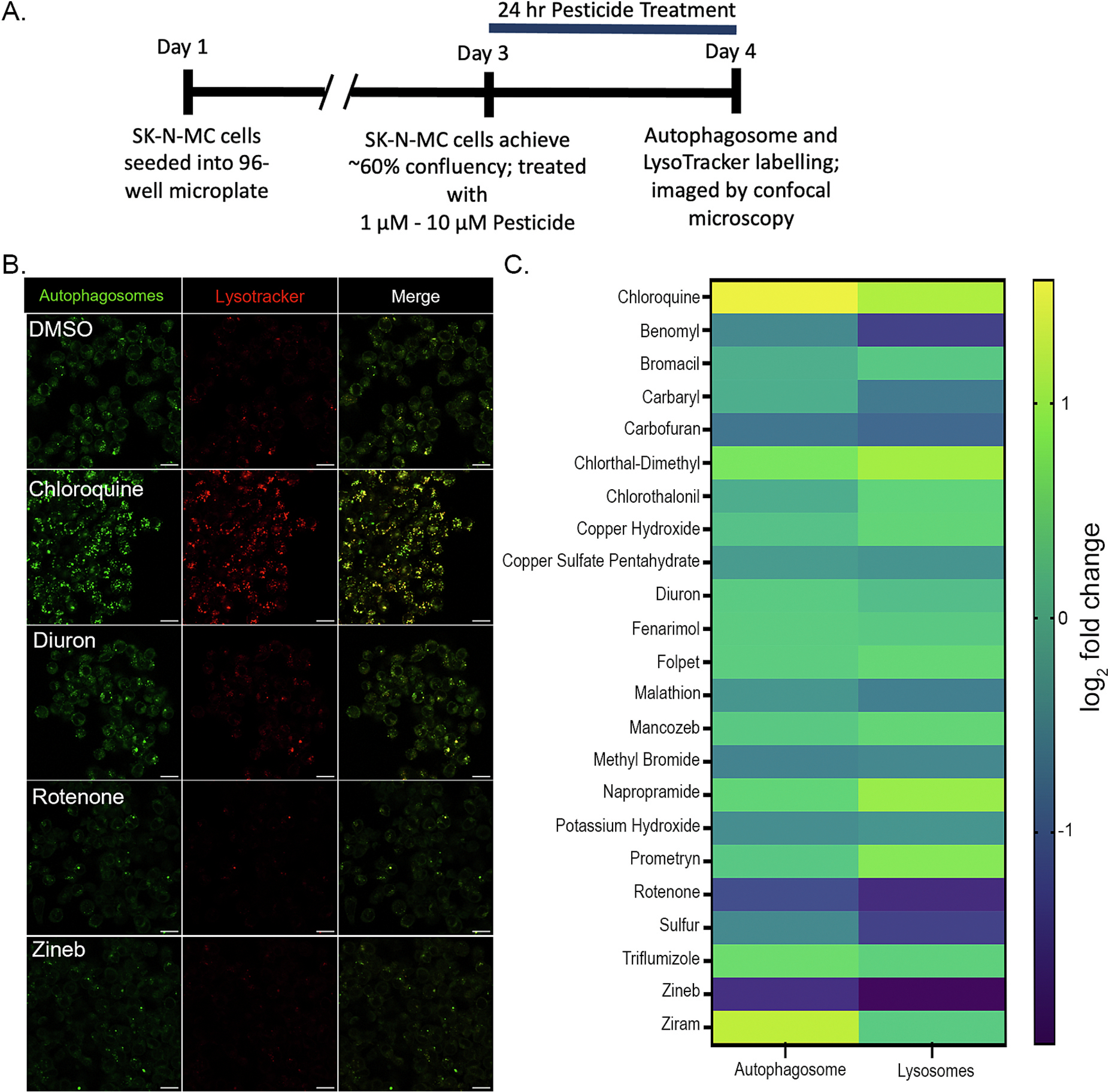
Screen for changes in autophagy components, autophagosomes and lysosomes, after pesticide exposure in SK-N-MC cells. A) Treatment schema outlining the treatment timeline from plate seeding, treatment, fixation, and dye-labelling. B) SK-N-MCs were treated pesticide for 24hr with the vehicle or pesticide, fixed, and rapidly labelled with Autophagosome Dye (green, Autophagy Detection Reagent) and Lysotracker (red, Lysotracker Red DND-99) for immediate live-cell imaging. Images shown are representative images for each condition. Scale bars: 25 μM. C) Twenty-two pesticides show significant perturbations (increased or decreased foci counts) to autophagosome and lysosome pools relative to vehicle and are considered hits. Data was analyzed as mean ± SEM and represented as log2 fold change from vehicle (0.1% DMSO); N = 3 images/well, 2 biological replicates with paired control wells. All pesticides were tested at 10 μM, except for chlorothalonil, mancozeb, and rotenone (1 μM), with a final vehicle concentration of 0.1% DMSO. Pair-wise comparisons were conducted by Student’s two-tailed T-test; raw foci count for each pesticide were compared to a paired-vehicle condition. The pesticide was considered a hit if a significant difference (P < 0.05) was observed for either the autophagosome or lysosome category. (For interpretation of the references to colour in this figure legend, the reader is referred to the web version of this article.)

All 62 pesticides were evaluated for their effects on autophagic components, autophagosomes and lysosomes, after a 24-hour exposure in SK-N-MC cells (Fig. 3A). The cytotoxicity of the panel of pesticides in SK-N-MC cells was determined empirically using a LIVE/DEAD dye assay with concentrations ranging from 0.1 μM – 30 μM. The treatment was well-tolerated up to 30 μM for most of the pesticides, evidenced by less than 25% cell death relative to control (Supplement Fig. 2). The 10 μM concentration was used for the autophagy assay in live SK-N-MC cells, except 1 μM was used for chlorothalonil, rotenone, and mancozeb due their toxicity at higher concentrations. Treatment with the autophagy inhibitor, chloroquine, was used as a positive control to show inhibition of autophagy demonstrated by the increase in autophagosome and lysosome puncta assessed by confocal microscopy (Fig. 3B). The screen revealed 16 pesticides that significantly altered the number of autophagosomes and 14 pesticides that significantly altered the number of lysosomes in SK-N-MC cells relative to vehicle for a total of 22 pesticide hits (Fig. 3C). While none of the pesticides achieved the high levels of both autophagosomes and lysosomes present with the autophagy inhibitor chloroquine, the pesticides chlorthal-dimethyl, napropamide, prometryn, and ziram all exhibited the largest increases in autophagosome and lysosome foci relative to vehicle (Fig. 3C). A subset of pesticides behaved in a similar pattern of moderate increases in both autophagosome and lysosome labeling (copper hydroxide, diuron, fenarimol, mancozeb, and triflumizole, Fig. 3C). A separate subset contributed to a reduction in autophagosome and lysosome labeling (benomyl, carbofuran, rotenone, sulfur, and zineb, Fig. 3C). Results for the full panel of screened pesticides through the autophagy assay can be found in Supplement Fig. 3 and Supplement Tables 2 and 3.

### 3.3. Pesticides with an increased odds ratio (OR) for PD in a case-control study of PD

**Fig. 4.**
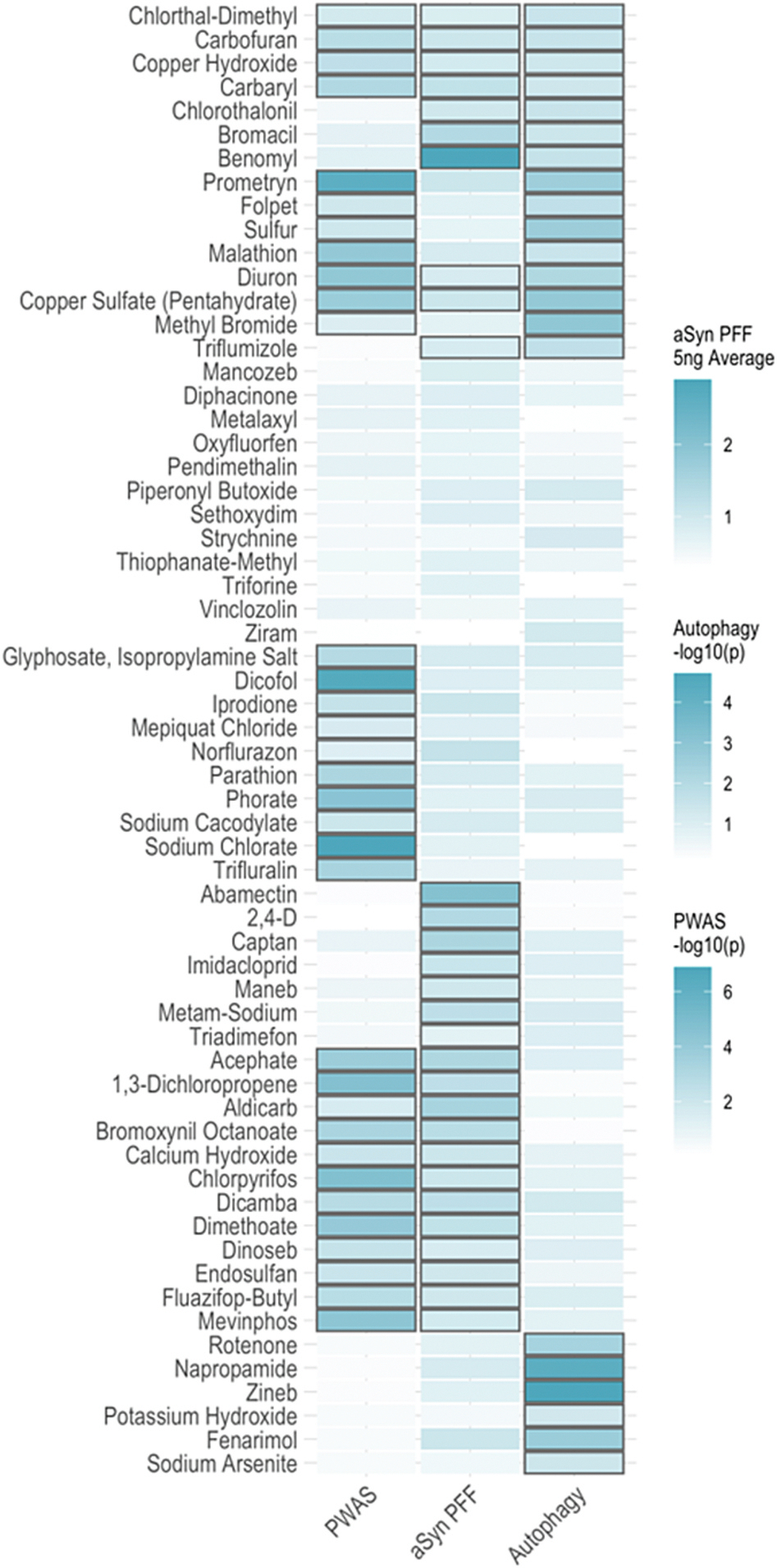
Six pesticides are identified as hits across all three screens. Carbaryl, carbofuran, copper sulfate pentahydrate, chlorthal-dimethyl, copper hydroxide and diuron are triple hits.

To further stratify and identify relevant pesticide exposures, we integrated the results from the 2 cell-based screens with the results from the untargeted screening of individual pesticides for association with PD (i.e., PWAS). The PWAS analysis tested 288 pesticides individually for association with PD in 1653 study participants, controlling for age, sex, race/ethnicity, education level, and index year (of diagnosis or interview) to account for temporal trends in pesticide use. Exposure was estimated at both the participant’s residence and workplace and associations were independently evaluated for each exposure location. Overall, 68 pesticides were linked to PD (25 at FDR < 0.01, 28 at 0.01 ≤ FDR ≤ 0.05, and 15 at 0.05 < FDR < 0.10) (Paul et al., 2023). By integrating the PWAS findings with the full panel of 62 pesticides screened in the cell-based assays, we found 34 pesticides had an association with PD, with sodium chlorate, kelthane (dicofol), and prometryn showing the largest OR for PD (Fig. 4). The 34 pesticides that were identified by the PWAS in our screen call attention to highly relevant real-world exposures. As the goal of this study was to identify pesticides using an AOP framework, the PWAS analysis was applied as a filter to the double positive hits from the cell-based assays to identify relevant exposures that could be evaluated empirically and epidemiologically. At this stage of prioritization in the pesticide screen, we next focused on pesticides that promoted α-syn transmission, altered autophagy, and were associated with PD for further investigation.

### 3.4. Triple-hits assessed for inducing aminergic neuron loss in ZF

**Fig. 5.**
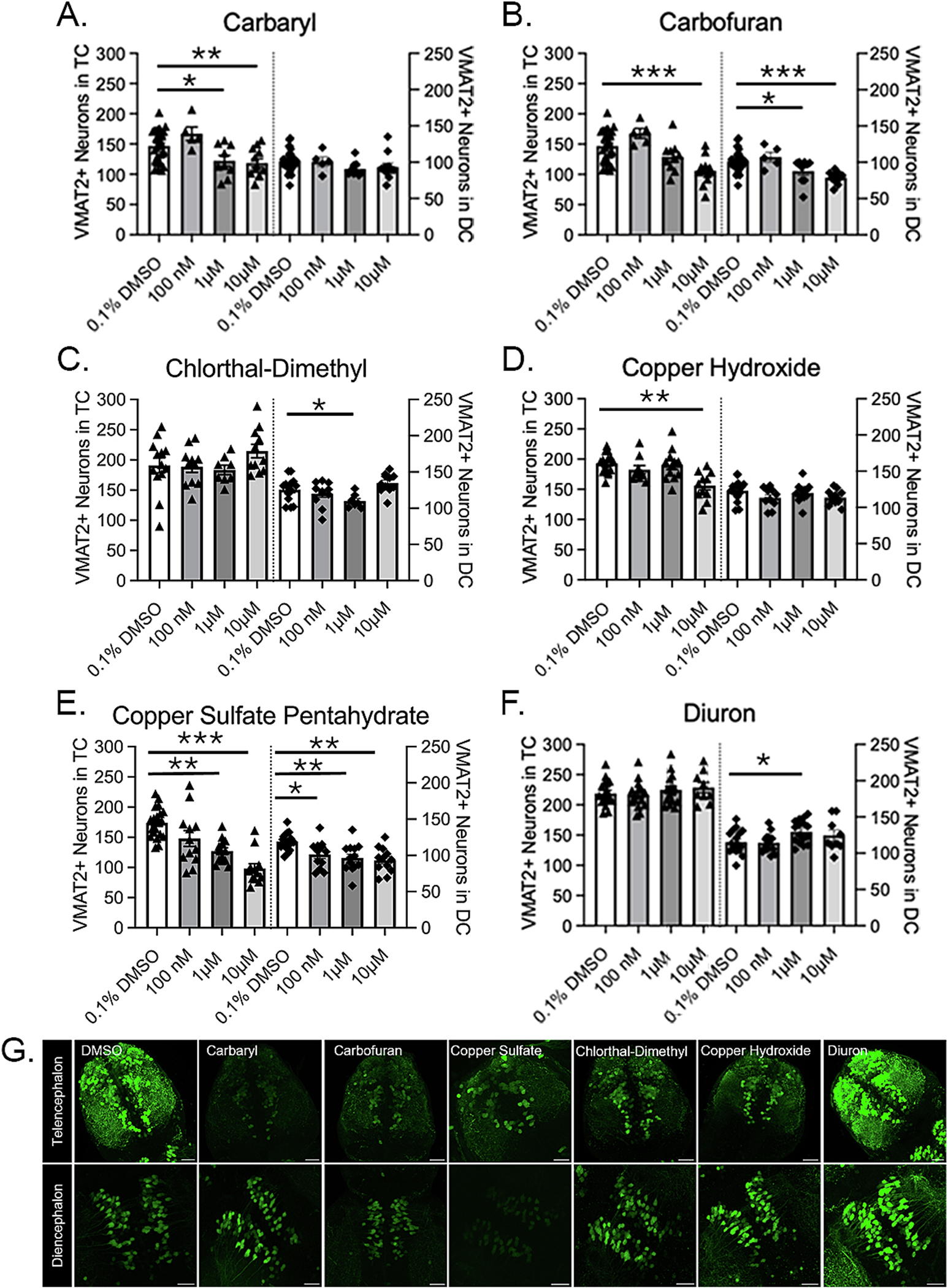
Carbaryl, carbofuran, copper hydroxide, and copper sulfate pentahydrate induce aminergic neuron loss in 7dpf zebrafish brain. The number of *VMAT2* + neurons in the ZF telencephalon (TC) and diencephalon (DC) were evaluated after pesticide exposure at multiple doses following a 6 day exposure period. The lowest dose at which we observed a significant reduction in the number of neurons is identified in each panel. A) Carbaryl reduced the number of *VMAT2* + neurons in the TC (at 1 μM), and showed no effect in the DC. B) Carbofuran reduced the number of *VMAT2* + neurons in both the TC and DC (at 10 μM and 1 μM, respectively). C) Chlorthal-Dimethyl reduced the number of *VMAT2* + neurons in the DC (only at 1 μM) but showed no effect in the TC. D) Copper hydroxide reduced the number of *VMAT2* + neurons in the TC (at 10 μM) and showed no effect in the DC. E) Copper sulfate pentahydrate (copper sulfate) reduced the number of *VMAT2* + neurons in both the TC and DC (at 0.1 μM). F) Diuron did not alter the number of *VMAT2* + neurons in the TC but showed a modest increase at only 1 μM in the DC. G) Representative images of ZF TC and DC brain regions for each condition at the highest concentration of pesticide evaluated (10 μM). Scale bars: 25 μM. Data shown as mean ± SEM; N = 5–14/condition. Dose response comparisons were conducted by one-way ANOVA with Dunnett’s post-hoc multiple comparisons test; all doses of pesticide were compared to vehicle (0.1% DMSO) alone. *P < 0.05, **P < 0.01, ***P < 0.001 Abbreviations: TC, telencephalon; DC, diencephalon; copper sulfate, copper sulfate pentahydrate.

In our screen framework, we hypothesized that the chemicals that were positive in all three screens would be the most likely to be causatively associated with increased PD risk. Thus, we first prioritized pesticides that that were hits in both cell-based screens by promoting α-syn PFF transmission and significantly altering autophagy, resulting in 10 pesticide candidates. We further filtered the 10 candidates through the PWAS findings and prioritized pesticide hits present in all three screens. While each of the screens resulted in several hits, our screen framework yielded 6 pesticide hits (i.e., triple hits, Fig. 4, Tables 1 and 2). To further test the triple hits for their ability to contribute to some of the pathology seen in patients, we utilized ZF as a model organism. ZF has emerged as a useful animal model to study neurodegenerative processes due to their optical clarity, rapid development, and conserved brain structures with mammals (Barnhill et al., 2020). The 6 pesticides that met the triple-hit criteria (i.e., carbaryl, carbofuran, copper sulfate, chlorthal-dimethyl, copper hydroxide, and diuron) were evaluated for aminergic neuron (*VMAT2* + ) toxicity. Significant *VMAT2* + neuron loss (P < 0.05) was observed with carbofuran (10 μM) and copper hydroxide (10 μM), carbaryl (1 μM), and copper sulfate (0.1 μM) in the telencephalon of 7 dpf ZF (Fig. 5A–G). Further, a significant loss of *VMAT2* + neurons (P < 0.05) was also observed in the diencephalon region with carbofuran (1 μM), chlorthal-dimethyl (1 μM), and copper sulfate (0.1 μM) in 7 dpf ZF (Fig. 5A–G). By applying a mechanism-based prioritization strategy, we identified 4 pesticides that contribute to aminergic neuron loss in ZF and provide support for the biological plausibility of these pesticide exposures.

## 4. Discussion

The association of pesticides with incident PD has been well established by many studies, but very few individual chemicals that confer PD risk have been described. Identification of individual toxicants is essential to determining whether the association is causative. There is no single accepted method to determine causality of associations, but the combination of epidemiological findings with cellular and animal studies has been employed to establish not only biological plausibility but also allow for causal inference. Here, we present empirical evidence that carbaryl, carbofuran, copper hydroxide, and copper sulfate implicates two AOPs associated with PD (promotion of α-syn transmission and alteration autophagy). These pesticides also contributed to a significant reduction in aminergic neuronal populations in our *in vivo* model. Using a mechanistic approach, we have identified 4 pesticides that have not been previously implicated in PD risk beyond our epidemiologic study with empirical evidence of molecular changes consistent with disease pathogenesis. These chemicals thus were identified not only as environmental risk factors for developing PD but exhibit considerable potential to alter AOPs critical to disease initiation and propagation. This study provides biological plausibility for the association between pesticide exposures and disease risk and highlights critical pathways that may link environmental exposures to disease processes. The findings of this study offer a strong basis for further investigation in additional mammalian models and epidemiological studies of PD.

As PD pathophysiology typically develops over decades in humans, an AOP approach is appropriate for identifying causative environmental toxicants. Since disruption of autophagy and α-syn transmission can lead to the development of PD over several years, applying the AOP framework as a screening tool is a strength and adds strong biological plausibility for these exposures and support causal inference. The integration of the PEG cohort data through the PWAS approach for identifying PD-associated pesticides is also a strength. Few cohorts have the ability to estimate subjects’ exposures to individual pesticides for over 45 years and not rely on subject recall for exposure assessment. Furthermore, the diagnosis of PD was made by a movement disorders specialist who followed them over time to further confirm an accurate case identification.

Despite the strengths of this study, we acknowledge there are limitations to this approach. While the AOP framework provides a strong foundation for mechanistic studies, it is likely that not all patients develop PD due to altered autophagy and increased α-syn transmission. Furthermore, the α-syn spread theory of progression is not universally accepted and there are limited experimental models that allow for detailed investigation. We also likely have missed some pesticides that contribute to risk since there are other relevant AOPs in PD. It is important to note that while this study isolates mechanisms of pathogenic α-syn transmission and autophagy, pesticide neurotoxicity is likely multifaceted and involves other well-established AOP key events in PD (e.g., mitochondrial dysfunction, neuroinflammation). Future work integrating these pathways will be essential for a holistic AOP assessment. Notwithstanding, we chose to improve our chances of identifying potential causative agents by using stringent criteria in our studies to increase the probability of identifying biologically relevant and plausible pesticide candidates.

Of the 6 triple hit pesticides, 4 of the pesticides contributed to aminergic neuron loss in zebrafish, but we did not detect a difference in the aminergic neuron population following exposure with chlorthal-dimethyl and diuron in 7 dpf ZF. While we did not detect neuron loss at the 7 dpf timepoint with chlorthal-dimethyl and diuron exposure, we cannot rule out altered molecular or protein interactions that may contribute to neuron loss with chronic exposure. In addition, despite the shared use-type classification of herbicide, these two chemicals are structurally distinct and do not appear to share a common mechanism of action. It is possible that factors related to chemical stability in aqueous solution or the metabolism of these chemicals in ZF may influence and drive chemical-specific neurotoxicity. With these considerations, our data suggest that more detailed investigation may be warranted to determine if chlorthal-dimethyl or diuron contribute to other neuropathological features related to PD. Another limitation of our study is the uncertainty of whether the selected concentrations in our experimental studies are relevant to human exposures. While this is extremely difficult to determine and must be considered in a pesticide-specific manner, we carefully considered actual use concentrations for these pesticides to estimate relevant human exposure levels. For example, based on the manufacturer’s instructions, 2 g of copper fungicide should be applied to 1 square meter, which would be estimated to be in the low millimolar range on the surface (Southern Ag – Liquid Copper Fungicide). In the case of copper sulfate, use is controlled for various purposes and can be applied in a concentration range of 0.25 ppm to 10 ppm on crops and in water which corresponds to a low micromolar range (approximately 1–––62 μM, US EPA Reg. No. 88633–3). Thus, to model likely environmentally relevant concentrations, we focused our investigation to low micromolar concentrations in our cell-based screens and observed reductions in *VMAT2* + neurons as low as 0.1 μM (copper sulfate) in 7 dpf ZF.

ZF have several advantages given that they develop rapidly with well-formed aminergic neurons, including dopaminergic neurons, in 3 days. ZF can be readily genetically modified to express fluorescent reporters, are transparent allowing for easy imaging of intact larvae, and they share a high homology with most mammalian genes (Barnhill et al., 2020). The means of ZF exposures is a potential limitation as the ZF in these studies were exposed whole-body to pesticide in an aqueous medium. However, we suggest that the whole-body exposure accounts for the three primary routes of exposure (inhalation, ingestion, and dermal) and may provide useful insights for pesticide prioritization ahead of exposure route-specific studies. In applying the developing ZF model to study a progressively neurodegenerative disease, the small size of developing ZF allows for multi-well format exposures to quickly and effectively screen for pesticide-induced neuronal toxicity (Hill et al., Jul 2005). In investigating the relationship between PD and pesticide exposure, these data support 4 prioritized triple-hit pesticides for in-depth mechanistic investigation, in addition to the hits for each individual *in vitro* screen.

In summary, we developed and implemented a multiscreen mechanistic approach to identify pesticides that increase the risk of developing PD and alter pathways critical to disease pathogenesis. We identified 4 pesticides that act to promote α-syn transmission and alter autophagy, are associated with PD in our epidemiologic analysis, and contribute to aminergic neuron loss in a ZF model. The results of this study offer a strong basis for further mechanistic studies of these pesticides in other PD experimental models to establish causality and develop potential treatments.

## Supplementary Material

MMC6

MMC7

MMC5

MMC4

MMC1

MMC3

MMC2

## Figures and Tables

**Table 1:** Multiscreen hits show alterations to autophagosome foci counts relative to vehicle in SK-N-MC cells. Autophagy assay results for autophagosome foci, with a paired vehicle condition, for each pesticide in the screen. Data shown as mean ± SEM, normalized to vehicle. Pair-wise comparisons were conducted by Student’s two-tailed T-test.

		Vehicle				Autophagosomes				
				
Concentration	Pesticide	Mean	±	SEM	N	Mean	±	SEM	N	P-Value

10 μM	Chloroquine	1	±	0.17	5	2.98	±	0.26	4	<0.01
10 μM	Carbaryl	1	±	0.14	3	1.02	±	0.08	4	0.92
10 μM	Carbofuran	1	±	0.08	3	0.60	±	0.10	4	0.03
10 μM	Chlorthal-Dimethyl	1	±	0.27	3	1.69	±	0.08	4	0.04
10 μM	Copper Hydroxide	1	±	0.06	3	1.20	±	0.10	6	0.23
10 μM	Copper Sulfate Pentahydrate	1	±	0.03	3	0.84	±	0.01	4	<0.01
10 μM	Diuron	1	±	0.05	3	1.30	±	0.05	4	0.01

**Table 2:** Multiscreen hits show alterations to lysosome foci counts relative to vehicle in SK-N-MC cells. Autophagy assay results for lysosome foci, with a paired vehicle condition, for each pesticide in the screen. Data shown as mean ± SEM, normalized to vehicle. Pair-wise comparisons were conducted by Student’s two-tailed T-test.

		Vehicle				Lysosomes				
				
Concentration	Pesticide	Mean	±	SEM	N	Mean	±	SEM	N	P-Value

10 μM	Chloroquine	1	±	0.23	5	2.18	±	0.32	4	0.02
10 μM	Carbaryl	1	±	0.08	3	0.62	±	0.10	4	0.04
10 μM	Carbofuran	1	±	0.15	3	0.54	±	0.10	4	0.05
10 μM	Chlorthal-Dimethyl	1	±	0.26	3	2.06	±	0.24	4	0.03
10 μM	Copper Hydroxide	1	±	0.20	3	1.42	±	0.08	6	0.04
10 μM	Copper Sulfate Pentahydrate	1	±	0.08	3	0.80	±	0.09	4	0.17
10 μM	Diuron	1	±	0.07	3	1.17	±	0.11	4	0.27

## Data Availability

All data are included in the manuscript and may be made available upon request.

## Appendix A. Supplementary data

Supplementary data to this article can be found online at https://doi.org/10.1016/j.envint.2026.110087.
