# Supplementary material for: Identification of pesticides associated with an increased risk of Parkinson’s disease using a multi-screen approach
Source: Environ Int. Author manuscript; Available in PMC 2026 Jul 27. (PMC13406333; doi:10.1016/j.envint.2026.110087)
Supplement: MMC6 [file NIHMS2191516-supplement-MMC6.docx]

Supplement Table 3

|  |  | **Vehicle** | | |  | **Lysosomes** | | |  |  |
| --- | --- | --- | --- | --- | --- | --- | --- | --- | --- | --- |
| **Concentration** | **Pesticide** | **Mean** | **±** | **SEM** | **N** | **Mean** | **±** | **SEM** | **N** | **P-Value** |
| 10 µM | 1,3-Dichloroproprene | 1 | ± | 0.14 | 3 | 0.89 | ± | 0.19 | 4 | 0.69 |
| 10 µM | 2,4-D | 1 | ± | 0.11 | 3 | 0.91 | ± | 0.12 | 4 | 0.61 |
| 1 µM | Abamectin | 1 | ± | 0.14 | 3 | 0.97 | ± | 0.06 | 6 | 0.81 |
| 10 µM | Acephate | 1 | ± | 0.19 | 4 | 0.68 | ± | 0.09 | 6 | 0.12 |
| 10 µM | Aldicarb | 1 | ± | 0.16 | 3 | 0.95 | ± | 0.03 | 4 | 0.72 |
| 10 µM | Benomyl | 1 | ± | 0.20 | 3 | 0.40 | ± | 0.07 | 4 | 0.03 |
| 10 µM | Bromacil | 1 | ± | 0.08 | 3 | 1.26 | ± | 0.06 | 5 | 0.04 |
| 10 µM | Bromoxynil Octanoate | 1 | ± | 0.07 | 3 | 1.10 | ± | 0.20 | 4 | 0.70 |
| 10 µM | Calcium Hydroxide | 1 | ± | 0.17 | 3 | 0.71 | ± | 0.10 | 4 | 0.18 |
| 10 µM | Captan | 1 | ± | 0.17 | 4 | 1.29 | ± | 0.07 | 5 | 0.12 |
| 10 µM | Carbaryl | 1 | ± | 0.08 | 3 | 0.62 | ± | 0.10 | 4 | 0.04 |
| 10 µM | Carbofuran | 1 | ± | 0.15 | 3 | 0.54 | ± | 0.10 | 4 | 0.05 |
| 10 µM | Chloroquine | 1 | ± | 0.23 | 5 | 2.18 | ± | 0.32 | 4 | 0.02 |
| 10 µM | Chlorthal-Dimethyl | 1 | ± | 0.26 | 3 | 2.06 | ± | 0.24 | 4 | 0.03 |
| 1 µM | Chlorothalonil | 1 | ± | 0.06 | 3 | 1.40 | ± | 0.10 | 5 | 0.03 |
| 10 µM | Chlorpyrifos | 1 | ± | 0.13 | 4 | 1.28 | ± | 0.23 | 4 | 0.33 |
| 10 µM | Copper Hydroxide | 1 | ± | 0.20 | 3 | 1.42 | ± | 0.08 | 6 | 0.04 |
| 10 µM | Copper Sulfate Pentahydrate | 1 | ± | 0.08 | 3 | 0.80 | ± | 0.09 | 4 | 0.17 |
| 10 µM | Dicamba | 1 | ± | 0.24 | 3 | 1.65 | ± | 0.16 | 6 | 0.06 |
| 10 µM | Dimethoate | 1 | ± | 0.11 | 3 | 0.97 | ± | 0.12 | 4 | 0.85 |
| 10 µM | Dinoseb | 1 | ± | 0.08 | 3 | 1.14 | ± | 0.07 | 5 | 0.25 |
| 10 µM | Diphacione | 1 | ± | 0.24 | 3 | 1.13 | ± | 0.40 | 5 | 0.83 |
| 10 µM | Diuron | 1 | ± | 0.07 | 3 | 1.17 | ± | 0.11 | 4 | 0.27 |
| 10 µM | Endosulfan | 1 | ± | 0.28 | 4 | 0.72 | ± | 0.08 | 4 | 0.37 |
| 10 µM | Fenarimol | 1 | ± | 0.10 | 3 | 1.29 | ± | 0.07 | 4 | 0.06 |
| 10 µM | Fluazifop Butyl | 1 | ± | 0.12 | 3 | 1.25 | ± | 0.06 | 4 | 0.09 |
| 10 µM | Folpet | 1 | ± | 0.06 | 3 | 1.45 | ± | 0.13 | 4 | 0.04 |
| 10 µM | Glyphosate Isopropylamine | 1 | ± | 0.24 | 3 | 0.59 | ± | 0.05 | 5 | 0.07 |
| 10 µM | Imidaclorpid | 1 | ± | 0.18 | 3 | 1.57 | ± | 0.20 | 5 | 0.11 |
| 10 µM | Iprodione | 1 | ± | 0.09 | 3 | 1.01 | ± | 0.08 | 4 | 0.97 |
| 10 µM | Kelthane | 1 | ± | 0.20 | 4 | 1.39 | ± | 0.12 | 4 | 0.15 |
| 10 µM | Malathion | 1 | ± | 0.01 | 3 | 0.66 | ± | 0.10 | 4 | 0.03 |
| 1 µM | Mancozeb | 1 | ± | 0.12 | 3 | 1.43 | ± | 0.29 | 4 | 0.28 |
| 10 µM | Maneb | 1 | ± | 0.14 | 4 | 1.23 | ± | 0.21 | 6 | 0.43 |
| 10 µM | Mepiquat Chloride | 1 | ± | 0.28 | 3 | 1.06 | ± | 0.22 | 4 | 0.87 |
| 10 µM | Metalyxl | 1 | ± | 0.06 | 3 | 0.88 | ± | 0.26 | 5 | 0.75 |
| 10 µM | Metam Sodium | 1 | ± | 0.46 | 3 | 1.51 | ± | 0.08 | 4 | 0.25 |
| 10 µM | Methyl Bromide | 1 | ± | 0.10 | 3 | 0.71 | ± | 0.10 | 6 | 0.10 |
| 10 µM | Mevinphos | 1 | ± | 0.19 | 3 | 0.71 | ± | 0.09 | 5 | 0.16 |
| 10 µM | Napropamide | 1 | ± | 0.05 | 4 | 1.97 | ± | 0.09 | 6 | <0.01 |
| 10 µM | Norflurazon | 1 | ± | 0.07 | 3 | 1.00 | ± | 0.03 | 5 | 0.99 |
| 10 µM | Oxyfluorfen | 1 | ± | 0.31 | 3 | 0.81 | ± | 0.16 | 4 | 0.58 |
| 10 µM | Parathion Methyl | 1 | ± | 0.28 | 3 | 1.59 | ± | 0.22 | 4 | 0.15 |
| 10 µM | Pendimethalin | 1 | ± | 0.14 | 3 | 1.16 | ± | 0.19 | 4 | 0.55 |
| 10 µM | Phorate | 1 | ± | 0.16 | 3 | 0.71 | ± | 0.09 | 7 | 0.12 |
| 10 µM | Piperonyl Butoxide | 1 | ± | 0.23 | 3 | 1.49 | ± | 0.07 | 4 | 0.06 |
| 10 µM | Potassium Hydroxide | 1 | ± | 0.21 | 3 | 0.80 | ± | 0.13 | 6 | 0.44 |
| 10 µM | Prometryn | 1 | ± | 0.16 | 3 | 1.81 | ± | 0.20 | 4 | 0.03 |
| 1 µM | Rotenone | 1 | ± | 0.14 | 4 | 0.34 | ± | 0.04 | 4 | <0.01 |
| 10 µM | Sethoxydim | 1 | ± | 0.11 | 3 | 1.15 | ± | 0.08 | 4 | 0.28 |
| 10 µM | Sodium Arsenite | 1 | ± | 0.21 | 4 | 1.57 | ± | 0.11 | 5 | 0.04 |
| 10 µM | Sodium Cacodylate | 1 | ± | 0.06 | 3 | 0.95 | ± | 0.15 | 5 | 0.82 |
| 10 µM | Sodium Chlorate | 1 | ± | 0.22 | 4 | 0.94 | ± | 0.15 | 5 | 0.82 |
| 10 µM | Strychnine | 1 | ± | 0.14 | 3 | 1.37 | ± | 0.09 | 4 | 0.07 |
| 10 µM | Sulfur | 1 | ± | 0.12 | 3 | 0.40 | ± | 0.04 | 4 | <0.01 |
| 10 µM | Thiophanate Methyl | 1 | ± | 0.23 | 3 | 0.71 | ± | 0.11 | 4 | 0.27 |
| 10 µM | Triadimefon | 1 | ± | 0.13 | 3 | 1.31 | ± | 0.23 | 4 | 0.34 |
| 10 µM | Triflumizole | 1 | ± | 0.13 | 3 | 1.36 | ± | 0.18 | 4 | 0.20 |
| 10 µM | Trifluralin | 1 | ± | 0.11 | 3 | 0.82 | ± | 0.06 | 4 | 0.19 |
| 10 µM | Triforine | 1 | ± | 0.13 | 3 | 1.00 | ± | 0.11 | 4 | 0.99 |
| 10 µM | Vinclozolin | 1 | ± | 0.01 | 3 | 1.47 | ± | 0.27 | 4 | 0.20 |
| 10 µM | Zineb | 1 | ± | 0.22 | 3 | 0.25 | ± | 0.02 | 4 | 0.01 |
| 10 µM | Ziram | 1 | ± | 0.19 | 5 | 1.30 | ± | 0.01 | 6 | 0.11 |

**Supplement Table 3. Results of lysosome foci differences in SK-N-MC autophagy assay.** Data shown as mean ± SEM, normalized to vehicle. Pair-wise comparisons were conducted by Student’s T-test; raw foci count for each pesticide condition were compared to a paired-vehicle condition. The pesticide considered a hit at P<0.05.
