# Supplementary material for: Identification of pesticides associated with an increased risk of Parkinson’s disease using a multi-screen approach
Source: Environ Int. Author manuscript; Available in PMC 2026 Jul 27. (PMC13406333; doi:10.1016/j.envint.2026.110087)
Supplement: MMC7 [file NIHMS2191516-supplement-MMC7.docx]

Supplement Figure Legends

**Supplement Figure 1. Effects of pesticides on transmission of pathogenic alpha-synuclein pre-formed fibrils in mouse primary hippocampal neurons. A)** Neuron viability was not affected by most pesticides and PFF treatment evaluated in the screen at 10 µM relative to the α-syn PFF-only condition as shown by NFL levels. **B)** Primary cultures treated with pesticides and α-syn PFFs were evaluated for phosphorylated α-syn (81A) immunofluorescence levels normalized to NFL levels and compared to PFF-only condition. Data shown as mean ± SEM (N=3) and is normalized to PFF-only condition.

**Supplement Figure 2. Dose response evaluation of pesticides on cell viability in human neuroblastoma SK-N-MC cells.** Dose response studies were conducted to evaluate pesticide-induced lethality. Most pesticides were found to be well-tolerated by SK-N-MC cells at 1µM - 10µM.

**Supplement Figure 3. Effects of pesticides on autophagosome and lysosome counts in SK-N-MC cells.** Data shown as log_2_ fold change relative to vehicle-treated cells following a 24-hour treatment with pesticide.
