# Supplementary material for: Identification of pesticides associated with an increased risk of Parkinson’s disease using a multi-screen approach
Source: Environ Int. Author manuscript; Available in PMC 2026 Jul 27. (PMC13406333; doi:10.1016/j.envint.2026.110087)
Supplement: MMC5 [file NIHMS2191516-supplement-MMC5.docx]

Supplement Table 2

|  |  | **Vehicle** | | |  | **Autophagosomes** | | |  |  |
| --- | --- | --- | --- | --- | --- | --- | --- | --- | --- | --- |
| **Concentration** | **Pesticide** | **Mean** | **±** | **SEM** | **N** | **Mean** | **±** | **SEM** | **N** | **P-Value** |
| 10 µM | 1,3-Dichloroproprene | 1 | ± | 0.02 | 3 | 0.92 | ± | 0.11 | 4 | 0.60 |
| 10 µM | 2,4-D | 1 | ± | 0.11 | 3 | 1.06 | ± | 0.08 | 4 | 0.68 |
| 1 µM | Abamectin | 1 | ± | 0.11 | 3 | 0.95 | ± | 0.05 | 6 | 0.66 |
| 10 µM | Acephate | 1 | ± | 0.13 | 4 | 0.83 | ± | 0.07 | 6 | 0.24 |
| 10 µM | Aldicarb | 1 | ± | 0.18 | 3 | 1.24 | ± | 0.13 | 4 | 0.32 |
| 10 µM | Benomyl | 1 | ± | 0.10 | 3 | 0.72 | ± | 0.05 | 4 | 0.04 |
| 10 µM | Bromacil | 1 | ± | 0.02 | 3 | 1.03 | ± | 0.03 | 5 | 0.50 |
| 10 µM | Bromoxynil Octanoate | 1 | ± | 0.11 | 3 | 1.01 | ± | 0.04 | 4 | 0.94 |
| 10 µM | Calcium Hydroxide | 1 | ± | 0.23 | 3 | 1.12 | ± | 0.01 | 4 | 0.57 |
| 10 µM | Captan | 1 | ± | 0.11 | 4 | 1.11 | ± | 0.08 | 5 | 0.41 |
| 10 µM | Carbaryl | 1 | ± | 0.14 | 3 | 1.02 | ± | 0.08 | 4 | 0.92 |
| 10 µM | Carbofuran | 1 | ± | 0.08 | 3 | 0.60 | ± | 0.10 | 4 | 0.03 |
| 10 µM | Chloroquine | 1 | ± | 0.17 | 5 | 2.98 | ± | 0.26 | 4 | <0.01 |
| 10 µM | Chlorthal-Dimethyl | 1 | ± | 0.27 | 3 | 1.69 | ± | 0.08 | 4 | 0.04 |
| 1 µM | Chlorothalonil | 1 | ± | 0.06 | 3 | 1.01 | ± | 0.07 | 5 | 0.95 |
| 10 µM | Chlorpyrifos | 1 | ± | 0.10 | 4 | 1.30 | ± | 0.16 | 4 | 0.16 |
| 10 µM | Copper Hydroxide | 1 | ± | 0.06 | 3 | 1.20 | ± | 0.10 | 6 | 0.23 |
| 10 µM | Copper Sulfate Pentahydrate | 1 | ± | 0.03 | 3 | 0.84 | ± | 0.01 | 4 | <0.01 |
| 10 µM | Dicamba | 1 | ± | 0.17 | 3 | 1.30 | ± | 0.09 | 6 | 0.13 |
| 10 µM | Dimethoate | 1 | ± | 0.04 | 3 | 1.17 | ± | 0.08 | 4 | 0.15 |
| 10 µM | Dinoseb | 1 | ± | 0.20 | 3 | 1.34 | ± | 0.09 | 5 | 0.11 |
| 10 µM | Diphacione | 1 | ± | 0.09 | 3 | 0.76 | ± | 0.12 | 5 | 0.20 |
| 10 µM | Diuron | 1 | ± | 0.05 | 3 | 1.30 | ± | 0.05 | 4 | 0.01 |
| 10 µM | Endosulfan | 1 | ± | 0.21 | 4 | 0.75 | ± | 0.03 | 4 | 0.28 |
| 10 µM | Fenarimol | 1 | ± | 0.04 | 3 | 1.32 | ± | 0.04 | 4 | <0.01 |
| 10 µM | Fluazifop Butyl | 1 | ± | 0.10 | 3 | 1.10 | ± | 0.04 | 4 | 0.33 |
| 10 µM | Folpet | 1 | ± | 0.07 | 3 | 1.32 | ± | 0.06 | 4 | 0.02 |
| 10 µM | Glyphosate Isopropylamine | 1 | ± | 0.15 | 3 | 0.70 | ± | 0.07 | 5 | 0.08 |
| 10 µM | Imidaclorpid | 1 | ± | 0.12 | 3 | 1.18 | ± | 0.09 | 5 | 0.27 |
| 10 µM | Iprodione | 1 | ± | 0.08 | 3 | 1.05 | ± | 0.03 | 4 | 0.51 |
| 10 µM | Kelthane | 1 | ± | 0.21 | 4 | 1.09 | ± | 0.16 | 4 | 0.75 |
| 10 µM | Malathion | 1 | ± | 0.06 | 3 | 0.81 | ± | 0.09 | 4 | 0.17 |
| 1 µM | Mancozeb | 1 | ± | 0.07 | 3 | 1.28 | ± | 0.19 | 4 | 0.27 |
| 10 µM | Maneb | 1 | ± | 0.17 | 4 | 0.69 | ± | 0.12 | 6 | 0.16 |
| 10 µM | Mepiquat Chloride | 1 | ± | 0.12 | 3 | 0.88 | ± | 0.09 | 4 | 0.46 |
| 10 µM | Metalyxl | 1 | ± | 0.08 | 3 | 0.97 | ± | 0.11 | 5 | 0.84 |
| 10 µM | Metam Sodium | 1 | ± | 0.29 | 3 | 1.60 | ± | 0.09 | 4 | 0.07 |
| 10 µM | Methyl Bromide | 1 | ± | 0.08 | 3 | 0.68 | ± | 0.02 | 6 | <0.01 |
| 10 µM | Mevinphos | 1 | ± | 0.07 | 3 | 0.88 | ± | 0.04 | 5 | 0.18 |
| 10 µM | Napropamide | 1 | ± | 0.13 | 4 | 1.42 | ± | 0.09 | 6 | 0.02 |
| 10 µM | Norflurazon | 1 | ± | 0.06 | 3 | 1.02 | ± | 0.04 | 5 | 0.81 |
| 10 µM | Oxyfluorfen | 1 | ± | 0.05 | 3 | 1.16 | ± | 0.14 | 4 | 0.41 |
| 10 µM | Parathion Methyl | 1 | ± | 0.29 | 3 | 1.26 | ± | 0.17 | 4 | 0.44 |
| 10 µM | Pendimethalin | 1 | ± | 0.06 | 3 | 1.21 | ± | 0.13 | 4 | 0.27 |
| 10 µM | Phorate | 1 | ± | 0.16 | 3 | 0.62 | ± | 0.11 | 7 | 0.08 |
| 10 µM | Piperonyl Butoxide | 1 | ± | 0.05 | 3 | 1.17 | ± | 0.08 | 4 | 0.14 |
| 10 µM | Potassium Hydroxide | 1 | ± | 0.13 | 3 | 0.75 | ± | 0.04 | 6 | 0.05 |
| 10 µM | Prometryn | 1 | ± | 0.05 | 3 | 1.26 | ± | 0.02 | 4 | <0.01 |
| 1 µM | Rotenone | 1 | ± | 0.20 | 4 | 0.44 | ± | 0.03 | 4 | 0.03 |
| 10 µM | Sethoxydim | 1 | ± | 0.13 | 3 | 1.05 | ± | 0.06 | 4 | 0.71 |
| 10 µM | Sodium Arsenite | 1 | ± | 0.07 | 4 | 1.36 | ± | 0.12 | 5 | 0.05 |
| 10 µM | Sodium Cacodylate | 1 | ± | 0.13 | 3 | 1.33 | ± | 0.10 | 5 | 0.09 |
| 10 µM | Sodium Chlorate | 1 | ± | 0.04 | 4 | 0.98 | ± | 0.09 | 5 | 0.86 |
| 10 µM | Strychnine | 1 | ± | 0.19 | 3 | 1.39 | ± | 0.13 | 4 | 0.14 |
| 10 µM | Sulfur | 1 | ± | 0.06 | 3 | 0.72 | ± | 0.03 | 4 | 0.01 |
| 10 µM | Thiophanate Methyl | 1 | ± | 0.12 | 3 | 0.98 | ± | 0.10 | 4 | 0.90 |
| 10 µM | Triadimefon | 1 | ± | 0.10 | 3 | 0.79 | ± | 0.06 | 4 | 0.11 |
| 10 µM | Triflumizole | 1 | ± | 0.15 | 3 | 1.55 | ± | 0.09 | 4 | 0.02 |
| 10 µM | Trifluralin | 1 | ± | 0.11 | 3 | 0.91 | ± | 0.06 | 4 | 0.46 |
| 10 µM | Triforine | 1 | ± | 0.03 | 3 | 1.02 | ± | 0.06 | 4 | 0.80 |
| 10 µM | Vinclozolin | 1 | ± | 0.23 | 3 | 1.63 | ± | 0.26 | 4 | 0.14 |
| 10 µM | Zineb | 1 | ± | 0.04 | 3 | 0.35 | ± | 0.02 | 4 | <0.01 |
| 10 µM | Ziram | 1 | ± | 0.17 | 5 | 2.35 | ± | 0.53 | 6 | 0.05 |

**Supplement Table 2. Results of autophagosome foci differences in SK-N-MC autophagy assay.** Data shown as mean ± SEM, normalized to vehicle. Pair-wise comparisons were conducted by Student’s T-test; raw foci count for each pesticide condition were compared to a paired-vehicle condition. The pesticide considered a hit at P<0.05.
