# Supplementary material for: Identification of pesticides associated with an increased risk of Parkinson’s disease using a multi-screen approach
Source: Environ Int. Author manuscript; Available in PMC 2026 Jul 27. (PMC13406333; doi:10.1016/j.envint.2026.110087)
Supplement: MMC4 [file NIHMS2191516-supplement-MMC4.docx]

Supplement Table 1

| **Pesticide** | **Vendor** | **Catalog Number** |
| --- | --- | --- |
| 1,3-DICHLOROPROPENE | Chem Service Inc | N-10193-500MG |
| 2-4-D | Sigma-Aldrich | 31518-250MG |
| ABAMECTIN | Chem Service Inc | N-10995-100MG |
| ACEPHATE | Sigma-Aldrich | 11-101-9093 |
| ALDICARB | Sigma-Aldrich | 33386-100mg |
| BENOMYL | Chem Service Inc | N-11138-100MG |
| BROMACIL | Chem Service Inc | N-11330-250MG |
| BROMOXYNIL OCTANOATE | Sigma-Aldrich | 11-101-9958 |
| CALCIUM HYDROXIDE | Sigma-Aldrich | 239232 |
| CAPTAN | Chem Service Inc | N-11400-250MG |
| CARBARYL | Sigma-Aldrich | 11-102-0173 |
| CARBOFURAN | Sigma-Aldrich | 11-102-0498 |
| CHLOROTHALONIL | Chem Service Inc | N-11454-250MG |
| CHLORPYRIFOS | Chem Service Inc | N-11459-100MG |
| CHLORTHAL-DIMETHYL | Chem Service Inc | N-11462-250MG |
| COPPER HYDROXIDE | Chem Service Inc | NG-I7032-1G |
| CUPRIC SULFATE PENTAHYDRATE | Sigma-Aldrich | C-7631 |
| DICAMBA, DIMETHYLAMINE SALT | Chem Service Inc | N-11656-250MG |
| DIMETHOATE | Sigma-Aldrich | 45449-100MG |
| DINOSEB | Chem Service Inc | N-11786-100MG |
| DIPHACINONE | Chem Service Inc | N-11793-100MG |
| DIURON | Sigma-Aldrich | 11-102-0532 |
| ENDOSULFAN | Chem Service Inc | N-10992-250MG |
| FENARIMOL | Chem Service Inc | N-11948-100MG |
| FLUAZIFOP-BUTYL | Chem Service Inc | N-11978-250MG |
| FOLPET | Sigma-Aldrich | 32057-250MG |
| GLYPHOSATE, ISOPROPYLAMINE SALT | Chem Service Inc | N-12134-250MG |
| IMIDACLOPRID | Chem Service Inc | N-12206-100MG |
| IPRODIONE | Chem Service Inc | N-12220-100MG |
| KELTHANE (DICOFOL) | Chem Service Inc | N-12290-100MG |
| MALATHION | Sigma-Aldrich | 11-102-0185 |
| MANCOZEB | Sigma-Aldrich | 45553-250MG |
| MANEB | Chem Service Inc | N-12355-250MG |
| MEPIQUAT CHLORIDE | Chem Service Inc | N-12371-100MG |
| METALAXYL | Chem Service Inc | N-12380-100MG |
| METAM SODIUM | Sigma-Aldrich | 45570-250MG |
| METHYL BROMIDE | Chem Service Inc | S-12417M8-1ML |
| MEVINPHOS | Chem Service Inc | NC1440273 |
| NAPROPAMIDE | Chem Service Inc | N-11585-250MG |
| NORFLURAZON | Chem Service Inc | N-12668-100MG |
| OXYFLUORFEN | Chem Service Inc | N-12742-100MG |
| PARATHION METHYL | Sigma-Aldrich | 11-101-9734 |
| PENDIMETHALIN | Sigma-Aldrich | 36191-100MG |
| PHORATE | Sigma-Aldrich | 11-101-7273 |
| PIPERONYL BUTOXIDE | Chem Service Inc | NC9954965 |
| POTASSIUM HYDROXIDE | Chem Service Inc | NG-I103-1G |
| PROMETRYN | Sigma-Aldrich | 11-101-9387 |
| ROTENONE | Sigma-Aldrich | R8875-1G |
| SETHOXYDIM | Chem Service Inc | N-13210-10MG |
| SODIUM ARSENITE | Chem Service Inc | NG-I121-1G |
| SODIUM CACODYLATE | Thermo Scientific Chemicals | AC214970100 |
| SODIUM CHLORATE | Thermo Scientific Chemicals | AC446411000 |
| STRYCHNINE | Chem Service Inc | N-13231-100MG |
| SULFUR | Sigma-Aldrich | 36576-250MG |
| THIOPHANATE METHYL | Sigma-Aldrich | 45688-250MG |
| TRIADIMEFON | Chem Service Inc | N-13636-500MG |
| TRIFLUMIZOLE | Sigma-Aldrich | 32611-100MG |
| TRIFLURALIN | Sigma-Aldrich | 45700-250MG |
| TRIFORINE | Chem Service Inc | N-13691-250MG |
| VINCLOZOLIN | Sigma-Aldrich | 45705-250MG |
| ZINEB | Sigma-Aldrich | 45707-250MG |
| ZIRAM | Chem Service Inc | N-13761-250MG |

**Supplement Table 1. Pesticide information.** Pesticide product information for pesticides in the screen.
