# Supplementary figures and images for: Identification of pesticides associated with an increased risk of Parkinson’s disease using a multi-screen approach
Source: Environ Int. Author manuscript; Available in PMC 2026 Jul 27. (PMC13406333; doi:10.1016/j.envint.2026.110087)

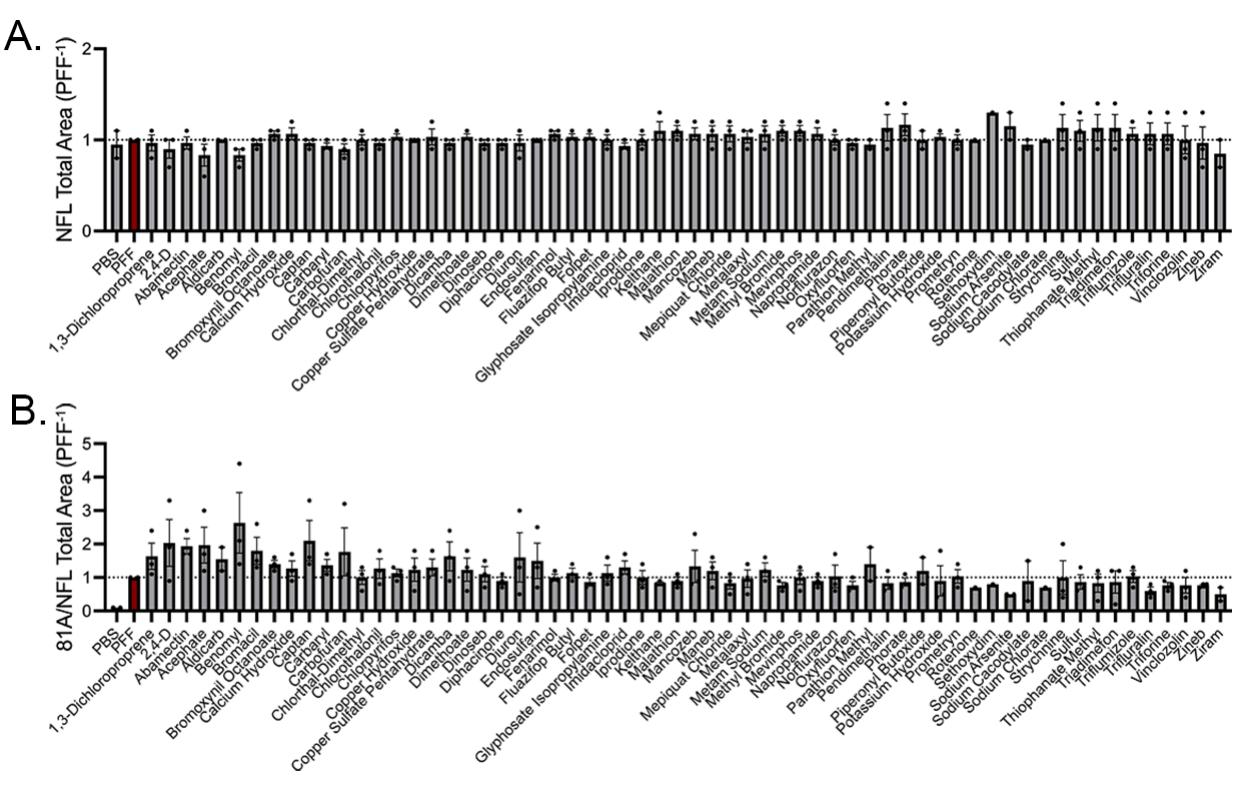

Supplement: MMC1 [file NIHMS2191516-supplement-MMC1.jpg]

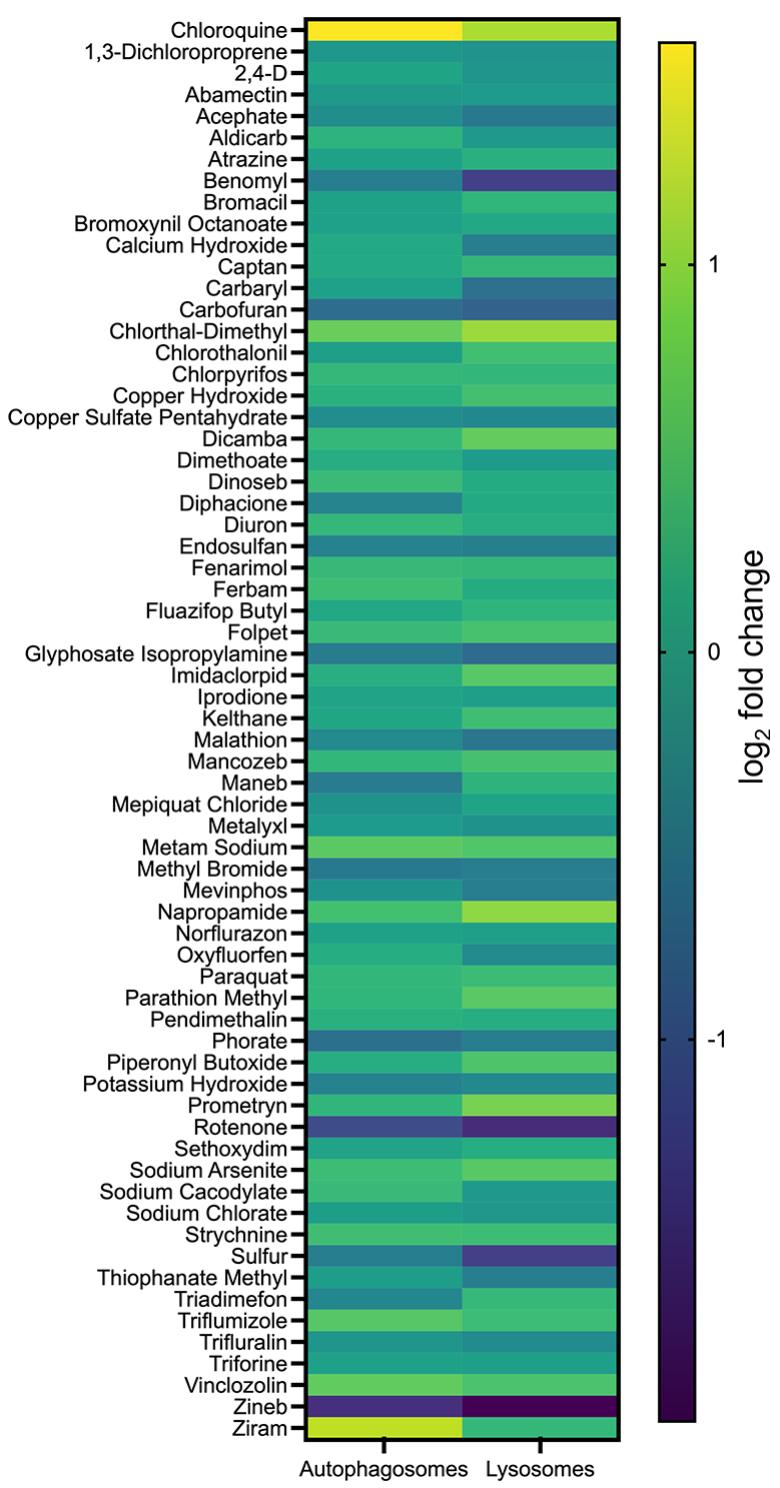

Supplement: MMC3 [file NIHMS2191516-supplement-MMC3.jpg]

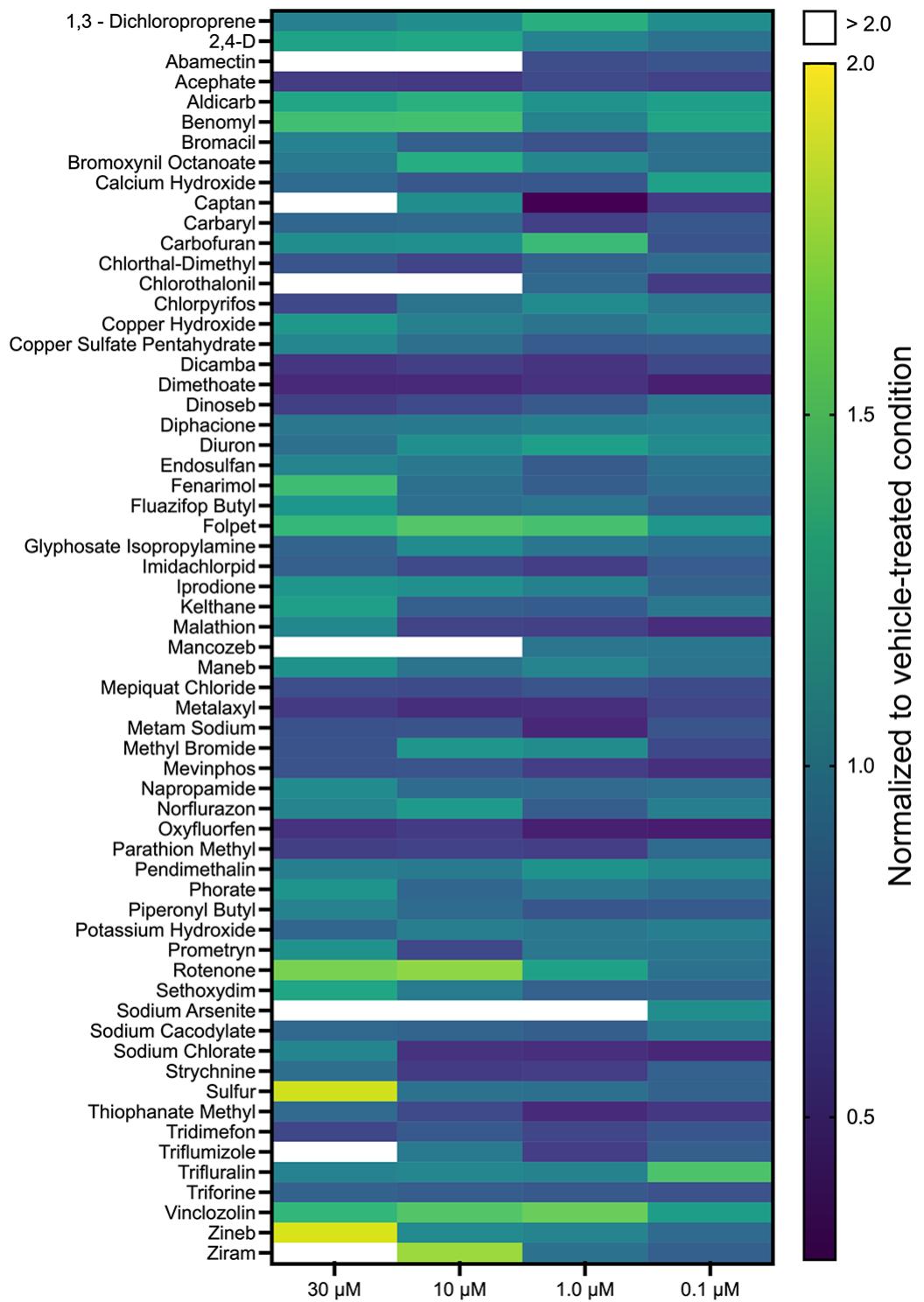

Supplement: MMC2 [file NIHMS2191516-supplement-MMC2.jpg]
